# Cholecystitis Due to Liver Fluke
*Report presented to Truro Medical Society on 1st February, 1949.


**Published:** 1949-07

**Authors:** G. P. O'Donnell

**Affiliations:** Surgeon, Redruth Hospital


### CHOLECYSTITIS DUE TO LIVER FLUKE*

BY

G. P. O'DONNELL.

*Surgeon, Redruth Hospital*

MRS. C. aged fifty, of Cornish origin, who had never lived outside England, was admitted to the Redruth-Camborne hospital suffering from severe recurring upper abdominal pain. For two years previously she had complained of vague epigastric discomfort and flatulence. For the past six months the pain had become colicky in character, increasing in severity and frequency and localized in the right hypochondrium.

On examination, there was well-defined tenderness with muscular guarding over the right upper rectus. X-ray showed a poorly functioning gall-bladder: no calculi were seen. Blood findings were leucocytes 16,000, otherwise normal (eosinophils 2 per cent). Diagnosis—Cholecystitis.

*Operation.* There were dense adhesions between the gall-bladder and the adjacent viscera. The gall bladder was normal in size, white and opaque. The walls were thickened by fibrous tissue and deposits of fat. There were two equally developed cystic ducts, one being continuous with the neck, the other arising a short distance above it. Each opened separately into the common duct. The gall bladder was removed. The common duct was dilated. No obstruction was found either from stone or stricture, nor was there any hardness or enlargement of the head of the pancreas. On exploration two living, fully developed liver flukes were removed from the upper end of the duct. Nothing abnormal was found in the distal end and the sound passed freely into the duodenum. The common duct was drained. She ran a normal course, and was discharged healed within three weeks. The bile and faeces were examined on several occasions whilst she was in hospital and again six weeks after discharge. No ova were ever found.

Before leaving hospital she was put on a course of emetine, one gram being given daily hypodermically for fourteen days. Since the operation she has not complained and at present seems quite well.

Cases of diagnosed parasitic infection due to liver fluke (Fasciola Hepatica) are rare. Up to 1945 Manson-Bahr recorded 150, mainly from Cuba. The other reported cases were from Argentine, Hungary, Germany, Switzerland, Greece, China and England.

* Report presented to Truro Medical Society on 1st February, 1949.

In England the fluke occurs most frequently in sheep, where it produces the disease known as "Liver Rot". It is also prevalent in cattle to a larger extent than was suspected. The average incidence in apparently healthy cattle, based on the examination of 73,000 slaughtered in June, 1942, was 17.3 per cent. The percentage was higher in the North than in Southern England.

The life cycle of the liver fluke is complicated. It lives in the bile ducts of the primary host, i.e. sheep, cattle and man; duration of life is stated to be probably about eighteen months. Each fluke can produce up to 50,000 eggs at once. These are excreted in the faeces and can live in water or damp grass for upwards of five months, before hatching with the consequent liberation of the miricidum—the first larval stage. The miricidum quickly perishes unless it finds the intermediate host, the water snail. In the snail it undergoes three further stages of development in six to seven weeks. On leaving the snail there is a final stage of development into the metacercaria. In this stage the larvae are very resistant. They can live in water for fourteen days; up to forty-eight days when completely dry; and in a refrigerator in a moist medium for eleven months.

The metacercaria is the infective stage for the primary host, being ingested in water or uncooked vegetables, e.g. water-cress. The larvae pass through the stomach unaltered. On reaching the small intestine the cyst corm is dissolved, freeing a young fluke. The young fluke bores through the intestinal walls into the abdominal cavity. It reaches the surface of the liver through the peritoneum, burrows through the fibrous capsule and the liver substance producing necrosis and finally comes to rest in the bile ducts. It has been suggested that at times it passes directly to the liver through the common duct.

On occasions it enters the blood-stream and comes to rest in different parts of the body, such as the brain or under the skin. If it lodges in any region except the liver the young fluke dies and undergoes calcification or forms a sterile abscess. In the bile ducts it becomes a mature hermaphroditic parasite in about three months and lays eggs, thus starting a new cycle.

*Symptoms.* In mild cases of infection there is slight gastro-intestinal disturbance, such as dyspepsia, alternating diarrhoea and constipation. In the more severe cases there is hepatic colic, due to mechanical irritation, acute cholecystitis or jaundice if the bile ducts become blocked. There are rigidity and tenderness of the abdominal walls during migration of the parasites. Frequently there is urticaria, as with other parasitic infections, due to sensitation to foreign proteins. There is persistent diarrhoea. The severe cases are accompanied by a leucocytosis up to 50,000, but usually in the region of 20,000. In the acute early cases this is due largely to an absolute increase in the eosinophils, which may be up to 60 per cent. and in part to liver damage and incidental infection.

Chronic cases develop anaemia which is due to two causes: primary toxaemia producing a secondary anaemia in the early stages, followed by macrocytic anaemia of cirrhosis in later stages. As the anaemia progresses, leucocytosis diminishes and there may be finally a leucopoenia. Boycott says the anaemia is due to toxic action causing aplasia of bone marrow. Wintrole ascribes it to hepatic insufficiency leading to lack of the "Anti-pernicious principle". Anaemia in fluke infection is differentiated from pernicious anaemia by uniformity of macrocytes; anisocytosis is not present, but there may be some poililocytosis. Achlorhydia is unusual.

Changes in the liver of infested animals are due to mechanical irritation and toxic action of the parasites. The superficial ducts are hypertrophied and sclerotic and the lumina are constricted, containing a dark substance consisting of muco-pus, ova, parasites and their faeces. In later chronic stages large convoluted cavities are present. These are formed by intercommunication of large bile ducts which become dilated owing to peripheral blockage. There is excess of fluid in the abdomen. The liver is small and sclerotic. If the animal dies in the acute stage, the liver is enlarged, haemorrhagic and friable. Usually blood-stained fluid is found in the abdomen. Large numbers of immature flukes can be expressed from the cut liver surface.

*Treatment.* Treatment should start on the farm, by clearing stagnant ditches, removing and burning the contaminated weeds and grass; and treating the land with copper sulphate. If the sheep are infected they are treated with carbon tetrachloride (1 mil. in capsules, monthly from October to April): in man give emetine injections, 0.5 cg. per kilo of body weight.

Anti-bodies are produced in the blood as with hydatid infection and can be detected with a skin test, using dried extract of the parasite. Some immunity has been conferred on rabbits by injecting increasing doses of dried parasites. This is still in the experimental stage, but may be of importance in prevention of infection in sheep.

